# Acute myeloid leukemia with new complex t(8;21;22) induced hemophagocytic lymphohistiocytosis: A case report

**DOI:** 10.1097/MD.0000000000012762

**Published:** 2018-11-02

**Authors:** Yuling Wu, Juan Xu, Kai Shen, Jie Ji, Chenlu Yang, Ting Liu, Bing Xiang

**Affiliations:** Department of Hematology, Hematology Research Laboratory, West China Hospital of Sichuan University, Chengdu, Sichuan, People's Republic of China.

**Keywords:** acute myeloblastic leukemia, chemotherapy, hematopoietic stem cell transplantation, hemophagocytic lymphohistiocytosis, variant translocation

## Abstract

**Rationale:**

The balanced translocation t(8;21;22)(q22;q22;q11.2) is not reported previously, although t(8;21)(q22;q22) is seen in approximately 7% of adults and most frequent abnormality in children with newly diagnosed acute myeloid leukemia (AML). AML-associated hemophagocytic lymphohistiocytosis (HLH) is a rare event, reported only of limited numbers. The present study reports a very rare case of t(8;21;22)(q22;q22;q11.2) with AML, not reported previously, and developed HLH at the same time.

**Patient concerns and diagnosis:**

A 15-year-old girl presented with a history of bleeding gums and high fever, leukocytosis, anemia, and thrombocytopenia. While waiting the result of bone marrow aspirate, the HLH-associated examinations were abnormal. Bone marrow aspirate showed a hypercellular marrow with 1% myeloblasts. The cytogenetic and molecular studies revealed the presence of abnormal karyotype-46, XX, t(8;21;22)(q22;q22;q11.2) and RUNX1–RUNX1T1 fusion gene. Genetic detections of HLH showed heterozygous genetic variants in lysosomal trafficking regulator (LYST). Hence, she was diagnosed with AML with t(8;21;22)(q22;q22;q11.2) and HLH.

**Interventions and outcomes:**

All HLH clinical symptoms disappeared after the 4 weeks treatment of HLH. Then the patient received standard AML induction chemotherapy and the leukemia relapsed after 2 cycles of high-dosed consolidation therapy. Eventually, the patient received emergent paternal haploidentical hematopoietic stem cell transplantation based on the complex variant translocation, leukemia replased state and HLH with compound heterozygotes mutation, and achieved sustained remission with RUNX1–RUNX1T1 negative for more than 1 year.

**Lessons:**

Patients with some specific recurrent cytogenetic abnormalities should be diagnosed with AML regardless of the blast count, for example t(8;21). We should improve the understanding of complex variant translocations. HLH-related genetic mutations were not only found in primary HLH, but also in second HLH.

## 1 Introduction

The World Health Organization (WHO) category “AML with recurrent genetic abnormalities” accounts for approximately 20%∼30% of AML cases.^[1]^ The balanced chromosomal translocation t(8;21)(q22;q22) is a frequent non-random cytogenetic abnormality in AML.^[2]^ In approximately 3% to 4% of AML patients, a complex variant translocation involving chromosomes 8, 21 and a third or fourth chromosome.

Hemophagocytic lymphohistiocytosis (HLH) is a rare but potentially life-threatening syndrome.^[3]^ Secondary HLH (S-HLH) is triggered by several causes, including infections, malignancies, metabolic diseases, autoimmune diseases, and acquired immune deficiencies. The malignancy-associated hemophagocytic lymphohistiocytosis is mostly associated with lymphoid neoplasms.^[4]^ Acute myeloid leukemia with HLH is rarely reported, only in some case reports.

Herein, we report a very rare case of t(8;21;22)(q22;q22;q11.2) with AML, and developed HLH. The complex variant is not reported in the previous literature and her initial bone marrow examination showed a low blast count.

## 2 Case report

**Figure 1:**
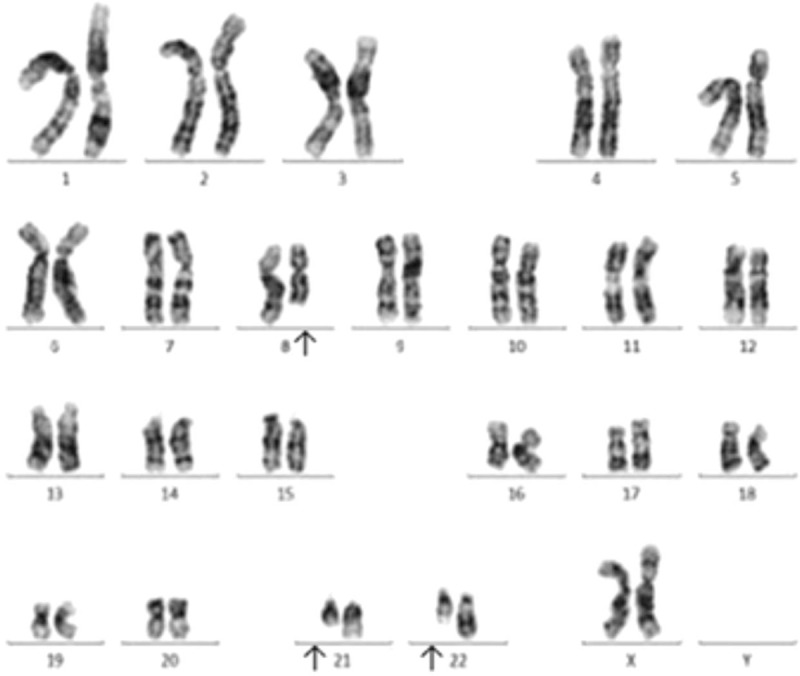
Chromosome karyotype analysis. There are 6 banded metaphases after cultured, and all show an abnormal karyotype-46, XX, t(8;21;22)(q22;q22;q11.2).

A 15-year-old girl was admitted into our center with a history of bleeding gums for 6 months and high fever for 18 days. On physical examination, spleen could be palpable below the costal margins without surperficial lymphadenopathy. The initial complete blood count revealed that the white blood cell count was 64.32 × 10^9^/L with 2% myeloblasts, hemoglobin level was 94 g/L, and the platelet count was 20 × 10^9^/L. Then bone marrow aspirate was taken on. She had a fever again and the hemogram gradually declined, while waiting the result of bone marrow aspirate. And we found that triglycerides (2.18 mmol/L), alanine aminotransferase (67 IU/L), aspartate aminotransferase (84 IU/L), lactate dehydrogenase (3537 IU/L), serum ferritin (81066 ng/mL) and soluble CD25 (1010 U/mL) were elevated. A reduced natural killer cell activity (12.5%) and fibrinogen level (0.5 g/L) were detected. Taken together with the clinical and laboratory findings, the patient was diagnosed with HLH according to the 2004 diagnostic guidelines for HLH.^[5]^ She was immediately treated with dexamethasone and etoposide based on the HLH-2004 regimen and dexamethasone dose was gradually reduced. Also bone marrow aspirate showed a hypercellular marrow with 1% myeloblasts. Flow cytometry (FCM) studies indicated that 1.9% of nucleated cells were positive for CD34, HLA-DR, CD13, CD33, CD56, CD117 and negative for CD5, CD7,CD16, CD19, which indicated an abnormal myeloid blast origin. Chromosomal analysis of the bone marrow cells showed an abnormal karyotype-46, XX, t(8;21;22)(q22;q22;q11.2) (Fig. 1). Moreover the RUNX1–RUNX1T1 fusion transcripts were detected in further molecular study. Other possible triggers of HLH were screened simultaneously and the genetic detections of HLH showed that the patient and her mother had the same heterozygous genetic variants in lysosomal trafficking regulator (LYST) (exon46; c.10526G >A; p.Arg3509Gln). Etiological examinations and autoimmune antibodies were negative.

Eventually, based on the presence of the recurrent genetic abnormality, the patient was diagnosed with AML with t(8;21;22)(q22;q22;q11.2), RUNX1–RUNX1T1. After 4 weeks of the treatment of HLH, the serum ferritin still elevated (684 ng/mL), but the HLH-associated clinical symptoms and other laboratory abnormalities disappeared. Repeated bone marrow examinations showed a hypercellular marrow with 13% myeloblasts and the RUNX1–RUNX1T1 fusion gene was still positive. Subsequently, the patient underwent standard AML induction chemotherapy consisting of cytarabine (160 mg, intravenous, days 1–7) and idarubicin (20 mg, intravenous, day 1, 10 mg, intravenous, days 2–3). The patient achieved complete remission (CR) with RUNX1–RUNX1T1 positive. Unfortunately she experienced leukemia replase revealed by 40.5% myeloblasts in the bone marrow after 2 cycles of consolidation chemotherapy consisting of high-dose cytarabine (4500 mg, intravenous, every 12 hours, days 1, 3, 5) and idarubicin (10 mg, intravenous, day 1). Then patient received standard CLAG (cladribine, cytarabine, granulocyte colony-stimulating factor) regimen, which was failed and there were still 59% myeloblasts in bone marrow. Finally patient received paternal haploidentical hematopoietic stem cell transplantation. After the transplantation, bone marrow achieved complete remission and RUNX1–RUNX1T1 fusion gene was negative for more than one year.

## 3 Discussion

According to the World Healthy Organization classification, patients with the specific recurrent cytogenetic abnormality of t(8;21)(q22;q22), should be diagnosed with AML regardless of the blast count.^[6]^ In the majority of cases, the RUNX1–RUNX1T1 fusion gene is the result of balanced translocation chromosomes 8 and 21.^[7]^ But there are some complex variant forms of translocations. Complex variants of t(8;21) was found to account for 3% to 4% of all t(8;21) cases.^[8]^ Those variants could involve chromosomes 1, 2, 3, 4, 5, 6, 7, 8, 10, 11, 12, 13, 14, 15, 16, 17, 18, 19, 20, or X.^[9–11]^ The t(8;21;22)(q22;q22;q11.2) in our case is a new form of complex variant reported nowhere before. Although the patients with t(8;21)(q22;q22) have a favorable prognosis, the prognostic impact of these complex variants is still unclear. Some studies hint that there are no obvious differences in complete remission and overall survival rate between the classical t(8;21) and the complex variants,^[12]^ but others indicate that the additional chromosome could change some biological characteristics of leukemic cells. For example, tumor cells may resist to the cytotoxic effects of chemotherapeutic agents.^[13]^ In our case, leukemia relapsed after a standard induction chemotherapy and high-dosed consolidation therapy. Thus we believe this complex variant may hint unfavorable prognosis and the significance needs further studies.

Malignancy-related HLH has been reported in many literatures and the most common malignancy types are hematological neoplasmas (93.7%) with NK- or T cell malignancies (35.2%), followed by B-cell lymphoma (31.8%), Hodgkin lymphoma (5.8%), acute leukemia (6.4%), and other hematologic neoplasms (14.4%).^[3]^ AML-associated HLH is only seen in some case reports without accurate data.

Genetic defects play an important role in familiar HLH (F-HLH) and childhood HLH, and are increasingly found in adult cases and S-HLH. These genes act in an autosomal recessive inheritance pattern, especially inheritance of a mutation at both alleles of a gene is required to manifest the disease; however, heterozygosity for an HLH mutation is occasionally found in an individual, and often associated with HLH-related diseases, including rheumatologic disorders, immunodeficiency, and others.^[14]^ This heterozygosity can be classified into 2 categories, including compound heterozygotes with a different mutation in each allele of the same gene and digenic inheritance with separate mutations in 2 different genes.^[15]^

Mutations in the LYST gene located on 1q42.1–42.2, have been identified as a cause of Chediak–Higashi syndrome that is associated with primary HLH,^[16]^ however, some cases who have LYST gene mutations never show any clinical manifestations of Chediak–Higashi syndrome.^[17]^ In the case, genetic testing of the patient and her mother identified a monoallelic mutation in LYST (exon46; c.10526G >A; p.Arg3509Gln) and we also found a heterozygous mutation on the intron of Exon 38 (c.9162 + 11G >A). The 2 mutations are novel mutations.

In the case, the patient was extensively investigated for the triggers of HLH, including infections, autoimmune deficiencies and malignancies, and the only finding was AML. Thus we thought AML was the trigger of HLH. The gene examination showed the same LYST gene mutation in the patient and her mother. Thus LYST gene monoallelic mutation in this patient may serve as the first hit and HLH will not have occurred without a second hit like AML in our case. Our study center is taking some research to confirm this double-hit hypothesis of HLH pathogenesis.

We are sorry that fluorescence in situ hybridization analysis is not performed to confirm t(8;21;22), because of lacking suitable probes and testing conditions. So the examinations of molecular genetics need further development in the future. The double-hit hypothesis of HLH pathogenesis has not been proved, and more research is needed to confirm it.

## Author contributions

**Conceptualization:** Jie Ji, Ting Liu, Bing Xiang.

**Investigation:** Kai Shen, Jie Ji, Chenlu Yang.

**Validation:** Yuling Wu, Juan Xu, Kai Shen.

**Writing – original draft:** Yuling Wu.

**Writing – review & editing:** Bing Xiang.

## References

[1] Arber DA Orazi A Hasserjian R The 2016 revision to the World Health Organization classification of myeloid neoplasms and acute leukemia. Blood 2016;127:2391–405. 27069254 10.1182/blood-2016-03-643544

[2] Grimwade D Hills RK Moorman AV Refinement of cytogenetic classification in acute myeloid leukemia: determination of prognostic significance of rare recurring chromosomal abnormalities among 5876 younger adult patients treated in the United Kingdom Medical Research Council trials. Blood 2010;116:354–65. 20385793 10.1182/blood-2009-11-254441

[3] Ramos-Casals M Brito-Zeron P Lopez-Guillermo A Adult haemophagocytic syndrome. Lancet 2014;383:1503–16. 24290661 10.1016/S0140-6736(13)61048-X

[4] Wang HL Xiong LX Tang WP A systematic review of malignancy-associated hemophagocytic lymphohistiocytosis that needs more attentions. Oncotarget 2017;8:59977–85. 28938698 10.18632/oncotarget.19230 PMC5601794

[5] Henter JI Horne A Arico M HLH-2004: diagnostic and therapeutic guidelines for hemophagocytic lymphohistiocytosis. Pediatr Blood Cancer 2007;48:124–31. 16937360 10.1002/pbc.21039

[6] Swerdlow SH Campo E Harris NL World Health Organization Classification of Tumours of Haematopoietic and Lymphoid Tissues. Lyon: IARC; 2008.

[7] Downing J The AML-ETO transcription factor in acute myeloid leukemia: biology and clinical significance. Br J Haematol 1999;106:296–308. 10460585 10.1046/j.1365-2141.1999.01377.x

[8] Minamihisamatsu M Ishihara T Translocation (8;21) and its variants in acute nonlymphocytic leukemia. The relative importance of chromosomes 8 and 21 to the genesis of the disease. Cancer Genet Cytogenet 1988;33:161–73. 3164243 10.1016/0165-4608(88)90026-x

[9] Vundinti BR Kerketta L Madkaikar M Three way translocation in a new variant of t(8;21) acute myeloid leukemia involving Xp22. Indian J Cancer 2008;45:30–2. 18453738 10.4103/0019-509x.40644

[10] Udayakumar AM Alkindi S Pathare AV Complex t(8;13;21)(q22;q14;q22)–a novel variant of t(8;21) in a patient with acute myeloid leukemia (AML-M2). Arch Med Res 2008;39:252–6. 18164974 10.1016/j.arcmed.2007.09.002

[11] Gmidene A Sennana H Frikha R An unusual three-way translocation t(21;8;1)(q22;q22;q32) in a case of acute myeloid leukemia (M2). Ann Biol Clin (Paris) 2012;70:213–6. 22484534 10.1684/abc.2012.0691

[12] Liu XP Xue YP Liu SH [An analysis of cytogenetic characteristics and prognosis of 189 t (8; 21) acute myeloid leukemia patients]. Zhonghua Nei Ke Za Zhi 2006;45:918–21. 17313880

[13] Gong JY Liu XP Li CW Clinical and laboratory study of a complex translocation t (6; 21; 8) (p22; q22; q22) in two patients with acute myeloid leukemia. Zhonghua Xue Ye Xue Za Zhi 2006;27:314–7. 16875580

[14] Spessott WA Sanmillan ML McCormick ME Hemophagocytic lymphohistiocytosis caused by dominant-negative mutations in STXBP2 that inhibit SNARE-mediated membrane fusion. Blood 2015;125:1566–77. 25564401 10.1182/blood-2014-11-610816 PMC4351505

[15] Zhang K Chandrakasan S Chapman H Synergistic defects of different molecules in the cytotoxic pathway lead to clinical familial hemophagocytic lymphohistiocytosis. Blood 2014;124:1331–4. 24916509 10.1182/blood-2014-05-573105 PMC4141517

[16] Barbosa MD Nguyen QA Tchernev VT Identification of the homologous beige and Chediak–Higashi syndrome genes. Nature 1996;382:262–5. 8717042 10.1038/382262a0 PMC2893578

[17] Mukda E Trachoo O Pasomsub E Exome sequencing for simultaneous mutation screening in children with hemophagocytic lymphohistiocytosis. Int J Hematol 2017;106:282–90. 28353193 10.1007/s12185-017-2223-3

